# Cytochrome P450 Reductase: A Harbinger of Diffusible Reduced Oxygen Species

**DOI:** 10.1371/journal.pone.0013272

**Published:** 2010-10-13

**Authors:** Kelath Murali Manoj, Sudeep Kumar Gade, Lazar Mathew

**Affiliations:** Heme-Flavin Laboratory, School of Bio Sciences and Technology, Center for Biomedical Research, VIT University, Vellore, Tamil Nadu, India; Indiana University, United States of America

## Abstract

The bi-enzymatic system of cytochrome P450 (CYP, a hemoprotein) and cytochrome P450 reductase (CPR, a diflavoenzyme) mediate the redox metabolism of diverse indigenous and xenobiotic molecules in various cellular and organ systems, using oxygen and NADPH. Curiously, when a 1∶1 ratio is seen to be optimal for metabolism, the ubiquitous CYP:CPR distribution ratio is 10 to 100∶1 or higher. Further, the NADPH equivalents consumed in these in vitro or in situ assemblies usually far exceeded the amount of substrate metabolized. We aimed to find the rationale to explain for these two oddities. We report here that CPR is capable of activating molecular oxygen on its own merit, generating diffusible reduced oxygen species (DROS). Also, in the first instance for a flavoprotein, CPR is shown to deplete peroxide via diffusible radical mediated process, thereby leading to the formation of water (but without significant evolution of oxygen). We also quantitatively demonstrate that the rate of oxygen activation and peroxide depletion by CPR accounts for the major reactivity in the CYP+CPR mixture. We show unambiguously that CPR is able to regulate the concentration of diffusible reduced oxygen species in the reaction milieu. These findings point out that CPR mediated processes are bound to be energetically ‘wasteful’ and potentially ‘hazardous’ owing to the unavoidable nature of the CPR to generate and deplete DROS. Hence, we can understand that CPR is distributed at low densities in cells. Some of the activities that were primarily attributed to the heme-center of CYP are now established to be a facet of the flavins of CPR. The current approach of modeling drugs to minimize “uncoupling” on the basis of erstwhile hypothesis stands questionable, considering the ideas brought forth in this work.

## Introduction

**Figure 1:**
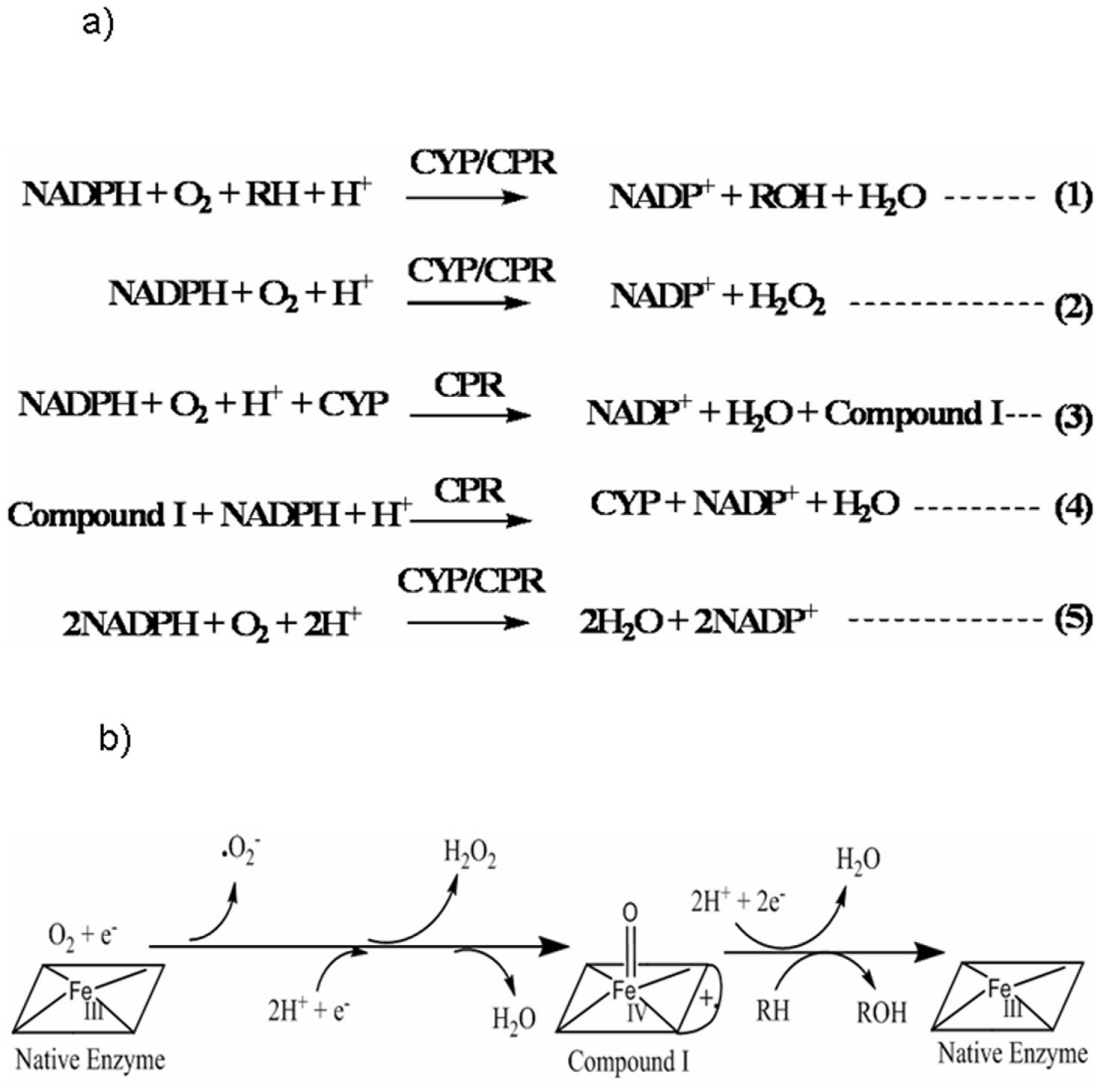
Hitherto held impressions of heme-centered phenomena in CYP+CPR reaction mixtures. ***1a: The reactions involved in CYP+CPR mixtures*** **.** ***1b:*** ** ***Erstwhile mechanism for DROS and water formation at heme center:*** The reactions above the straight arrows are uncoupled where as the ones below is the coupled/‘normal’ course.

Cytochrome P450s (CYPs), a family of diverse heme-thiolate proteins, metabolize several indigenous molecules and xenobiotics *in vivo* [1], [2]. CYPs also hold immense green chemistry potentials owing to their ability to selectively oxidize relatively non-reactive moieties and to generate chiral synthons [3], [4]. For their variety of oxidative reactions, the CYPs work in tandem with a highly conserved cytochrome P450 reductase (CPR, a diflavoenzyme) [5], [6]. The terminal redox equivalents are obtained from reduced nicotinamide adenine dinucleotides (NADPH and NADH, the former being preferred) and molecular triplet oxygen serves as the primary oxidant. The overall CYP+CPR reaction is also found to be “uncoupled” (Figure 1a and 1b), leading to the production of diffusible reduced oxygen species (DROS) of superoxide and peroxide in the reaction system [7]–[10]. Hitherto, DROS formation has been attributed to an uncoupling phenomenon supposed to occur at the heme-center [7], [9]–[11]. A binding of suitable substrate at the active site of CYP was shown to increase the redox potential of the heme iron, which is obligatory for facilitating the reduction of CYP by CPR through a protein-protein interaction [2], [12]. This coupling phenomenon is demonstrated and exemplified by camphor binding to the classical *Pseudomonad* CYP101 [13].

Since “uncoupling” leads to the loss of NADPH redox equivalents, it is hitherto understood to be a wasteful process [14]. Theoretically, one molecule of NADPH could give rise to one molecule of peroxide (Figure 1a, equation 2) or one molecule of hydroxylated product, via an overall two-electron process. However, it is experimentally seen by several workers that the amount of NADPH consumed, many times, far exceeds the total amounts of hydroxylated product and DROS formed in the reaction medium. To explain this stoichiometric imbalance, once again a heme-center based mechanism was proposed in which a two electron deficient oxygenated CYP catalytic intermediate, called the Compound I, consumes yet another molecule of NADPH (with the aid of CPR), giving rise to water [1], [2], [7]–[11], [15]. This is shown in Figure 1a, where equations 3 & 4 added give equation 5. Figure 1b depicts the overall mechanistic flowchart for the hitherto held understanding of coupled and uncoupled processes.

Curiously, when a 1∶1 ratio is seen to be optimal for metabolism [6], the ubiquitous CYP:CPR distribution ratio is 10 to 100∶1 or higher [7]. For these and some other reasons (detailed in the initial part of the discussion of this communication), we found the hitherto held hypotheses unconvincing, which forced us to explore the CYP+CPR bi-enzymatic system with a new perspective. This report brings forth both forgotten and novel facets of CPR to afford an alternate explanation for the variations of DROS in reaction milieu and the loss of redox equivalents in the CYP reaction mixture.

## Results

### Peroxide production by CPR

Tables 1 & 2 depict production of peroxide and the rate of oxidation of NADPH and NADH respectively. NADPH is known to be a better redox equivalent supplier than NADH in the CYP+CPR catalyzed hydroxylation reactions. It is seen that for oxygen activation by CPR, the same paradigm also holds true, as evidenced by lower NADH consumption rate and peroxide formation in Table 2 (in comparison to the values quoted in Table 1). In both these systems, significant amount of reductant consumption and peroxide production is seen with CPR alone, which is comparable with the amounts present when CYP and its substrate are introduced. Also, the overall magnitude of peroxide production or the rate of reductant depletion positively correlated to the concentration of CPR alone (for early time frames). It is seen that both peroxide production rate and total peroxide amount may reduce in time (Table 2).

**Table 1:** *Generation of peroxide by CPR from molecular oxygen and NADPH* in a pure reconstituted system is presented.

*Samples*	*Peroxide in milieu (µM)*			*NADPH consumption* (*µM/min.*)
	10 min.	20 min.	30 min.	
**25 nM CPR**	0.43±0.32	1.39±0.01	1.60±0.20	0.354
**100 nM CPR**	1.96±0.10	3.85±0.30	5.74±0.41	0.740
**400 nM CPR**	6.69±0.75	12.11±0.36	13.20±0.44	2.765
**100 nM CPR + 100 nM CYP + 200 µM Diclof.**	3.44±1.02	4.82±0.23	6.23±0.09	0.981

**Table 2:** *Generation of peroxide by CPR from NADH* and its correlation to depletion of NADH in a pure reconstituted system is presented.

*Samples*	*Peroxide in milieu (µM)*		*NADH consumption* (*µM/min.*)
	16 min.	35 min.	
**20 nM CPR**	0.45±0.4	0.31±0.30	0.04±0.006
**100 nM CPR**	0.70±0.67	1.06±0.10	0.10±0.002
**500 nM CPR**	3.48±0.75	2.81±0.44	0.49±0.001
**100 nM CPR + 100 nM CYP + 200 µM Diclof.**	2.08±1.04	2.03±0.48	0.37±0.009

### Peroxide depletion by CPR

**Figure 2:**
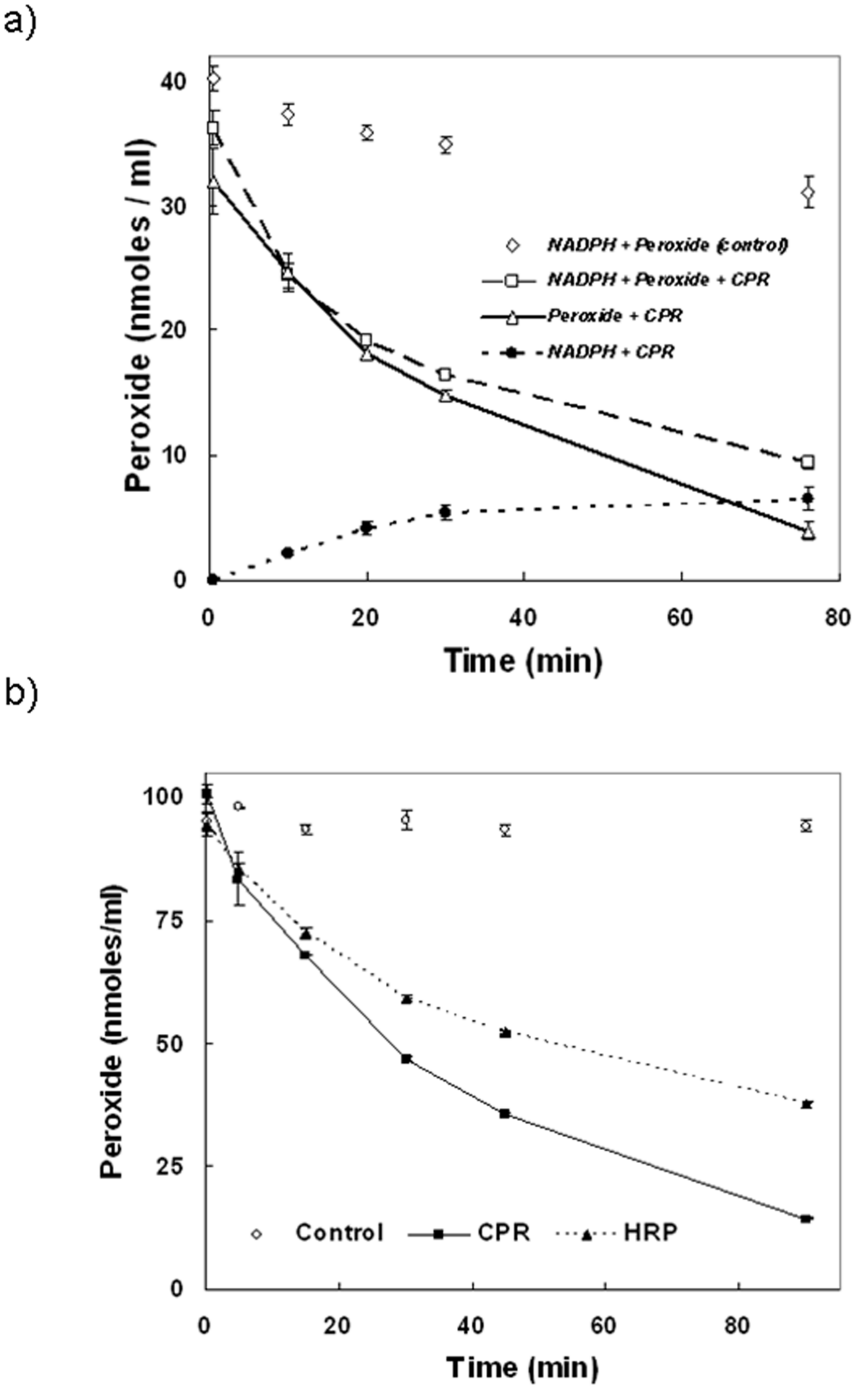
Characterizing the oxygen activation and peroxide depleting roles of CPR. **2a:** ***The peroxide depleting nature of*** *CPR* is depicted. The trace (triangles) shows that CPR does not need NADPH for depleting peroxide. The initial concentration of components were- (CPR)  = 230 nM, (NADPH)  = 164 µM & (H_2_O_2_)  = 40 µM. **2b:** ***Comparison of CPR's peroxide depleting function with an approximately equal concentration of HRP*** is shown. Catalase (160 nM) depleted peroxide to non-detectable levels within the first few seconds of incubation itself and hence, the trace is not shown. Initial concentrations were- (CPR)  =  (HRP)  = 160 nM, (H_2_O_2_)  = 100 µM.

Figure 2a shows the novel ability of mammalian CPR to deplete peroxide. Similar activities were exhibited by both rabbit and human CPR (results not shown). This observation indicates that CPR could also function as an effective peroxide scavenger. It could also be seen that CPR functions in equilibration of NADPH and peroxide. From preliminary studies, it was noted that initial rate of this peroxide depleting activity increased with the amount of initial enzyme or peroxide concentration in the milieu. However, the kinetics did not obey a simple Michaelis-Menten type hyperbolic profile (results not shown).

### Comparison of CPR's peroxide depletion activity with other enzymes

**Figure 3:**
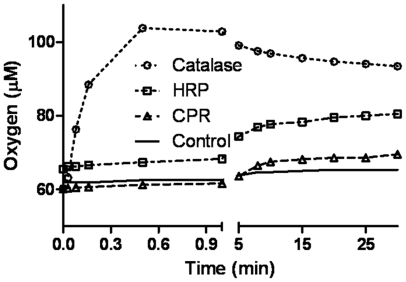
Tracing of oxygen evolution in peroxide depletion reactions.

A comparison in Figure 2b shows that CPR is akin to horseradish peroxidase (HRP) in its profile for depleting peroxide in the milieu. It is, however, more efficient than HRP at lower concentrations of peroxide. An equal amount of catalase depleted the peroxide to non-detectable limits within the first minute (results not shown). Incorporation of up to 3 equivalents (∼500 nM) of human CYP2C9 could not mediate any significant conversion of peroxide to water within an hour of incubation (results not shown). Figure 3 shows that CPR showed little elevation in dissolved oxygen concentration (quite akin to the negative control employed, met-myoglobin), whereas a classical peroxide dismutator like catalase (a positive control) did. HRP, which shows ability to utilize both peroxide and superoxide, showed a relatively lower elevation of oxygen concentration, in comparison to catalase.

### Involvement of radical reactions in CPR mediated depletion of peroxide

**Figure 4:**
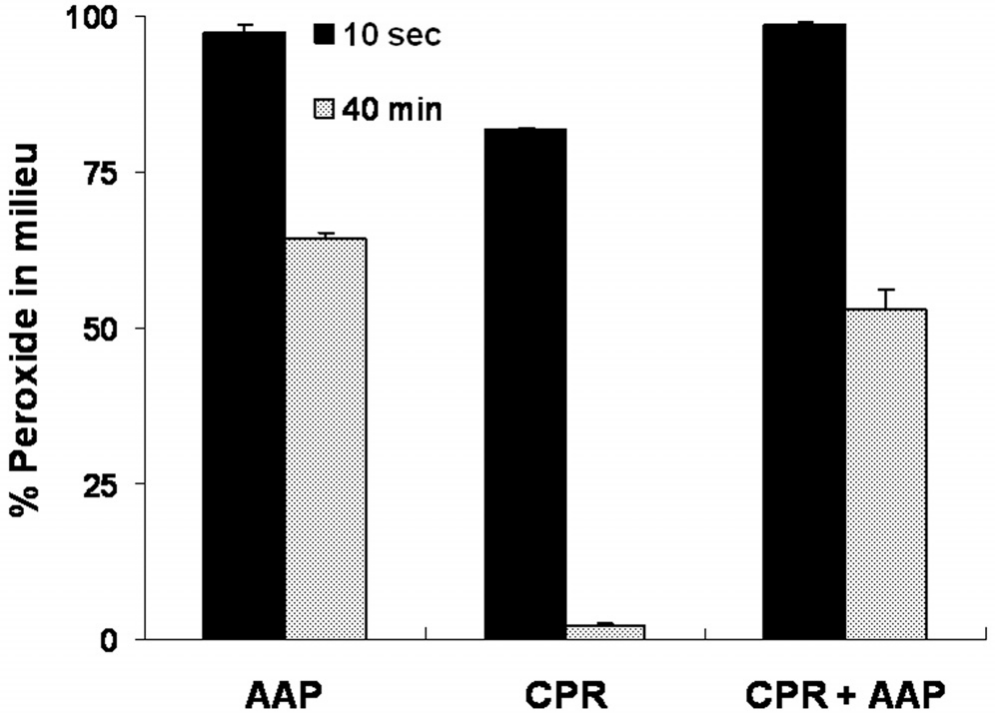
The effect of a radical scavenger in CPR mediated peroxide depletion.

Figure 4 shows the effect of inclusion of ascorbic acid palmitate (AAP), an efficient radical scavenger, on CPR mediated peroxide degradation. A positive control reaction with CPR showed that more than 97% of the initial peroxide was degraded in 40 minutes. A control with only AAP gave ∼35% of background peroxide degradation, whereas the test reaction including both CPR and AAP gave 47% degradation in the same period. Taking into account the autocatalytic degradation of peroxide, we see that the AAP containing CPR reaction mixture only gave ∼19% activity of the CPR containing reaction. This result positively indicates that the CPR mediated depletion of peroxide involves diffusible radicals.

## Discussion

To explain for the loss of NADPH equivalents, some workers had mooted the idea of water formation at the heme-center of CYPs [1], [2], [7]–[11], [15]. We chose to look at the CYP+CPR reaction system with a fresh perspective because three arguments were in strong opposition to such hypothesis-

1. It seeks conserved electron transfer machineries in a diverse array of CYPs but proton shuttling systems are not evident within the hydrophobic active site pockets of CYPs with elucidated crystal structure (like CYP2C9) [16], [17].
2. From preliminary investigations, one of us had seen that CYP2C9 and several other liver microsomal CYPs could not mediate the breakdown of peroxide on its own, to any noticeable extent. One would find it difficult to imagine that an iron-peroxo intermediate purported to be formed en route to Compound I normally, could not form (even to small amounts!) the catalytic intermediate required for peroxide depletion and water formation in the CYP + peroxide setup.
3. It seems highly unlikely that the wide variety of CYPs' highly electrophilic Compound I radical would “opt” for the relatively slower two-electron and two-proton transfers to form water. If the hitherto held hypothesis were operative, then CYPs' Compound I should have been easily isolated, like that of the peroxidases [18]. But in spite of several decades of efforts, the hunt for Compound I from microsomal CYPs is unfruitful.

Discovery of CPR's novel peroxide depletion function (and rediscovery of CPR's oxygen activation) now affords us an effective alternate hypothesis, where CPR is the more pivotal role-player in DROS production and regulation.

**Figure 5:**
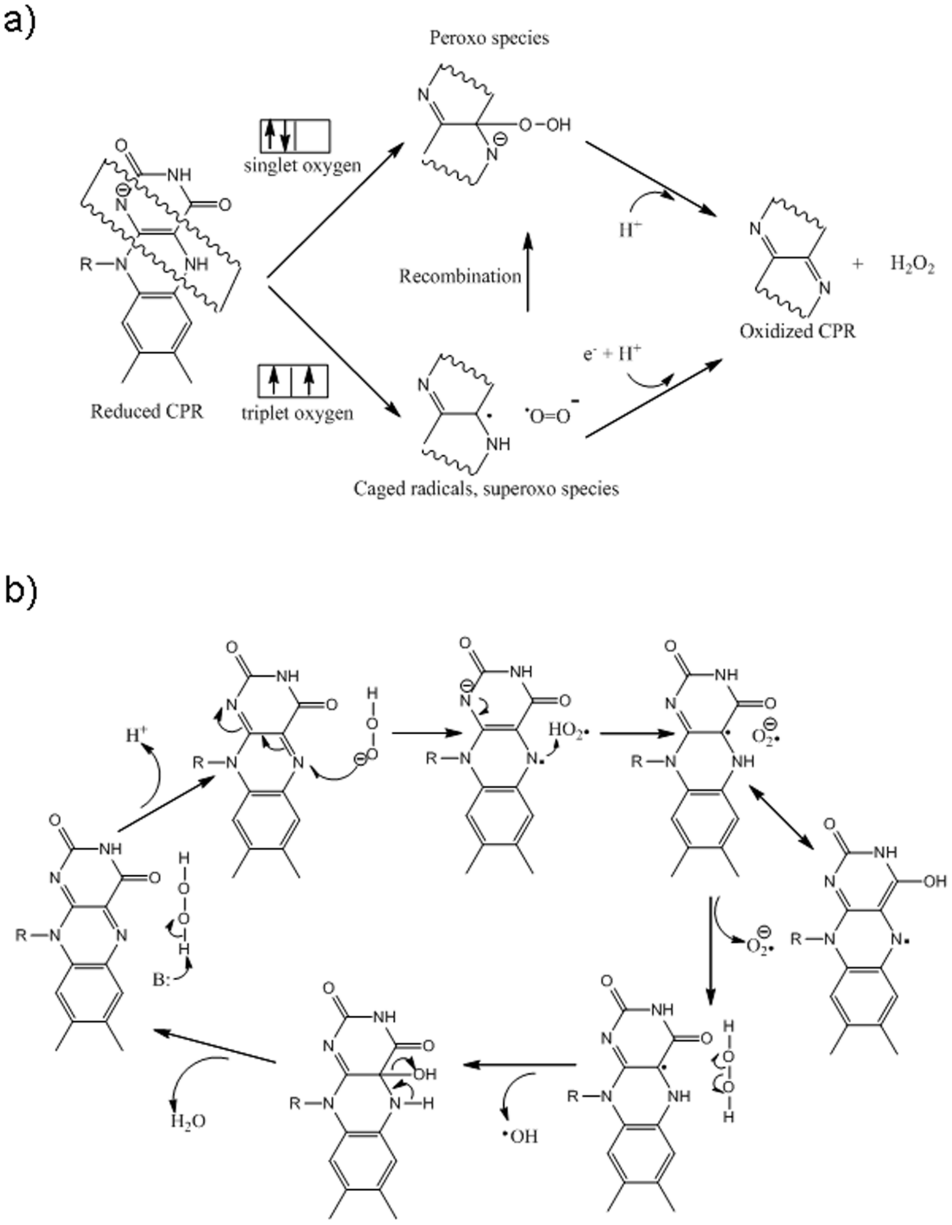
Molecular mechanisms of oxygen activation and peroxide depletion by CPR. ***5a. Activation of oxygen by reduced CPR to generate DROS. 5b: Probable flavin-based mechanistic route for degradation of peroxide***.

It was held for long that only catalysts employing transition metal elements with unpaired *d* electrons would be capable of activating the ground state oxygen. This was because “normal” diatomic (molecular) oxygen is in triplet state and its reaction with a singlet (which flavin is in!) to finally give a singlet product is essentially a spin forbidden process. In the 1970s, Steven Aust's research had originally shown that cytochrome c reductase (the erstwhile name for CPR, isolated with a tryptic cleavage of its hydrophobic N terminus) generated DROS in reaction milieu [19], [20]. Even Aust had subsequently conceded to peer-pressure that this process was an experimental artifact, probably resulting out of trace iron contaminations (SD Aust, personal communication to KMM, 2006). Through 1980s and 1990s, it was established clearly that reduced flavins could activate ground state molecular oxygen on their own merit, at various rates, depending upon the environment that the flavins were incorporated into the enzyme [21]–[23]. The significance of this aspect of flavin catalysis was subsequently disregarded while considering the overall mechanistic chemistry and stoichiometry of CYP + CPR reactions [7], [9]–[11]. The observations presented in Tables 1 and 2 provide quantitative confirmation that CPR is an efficient reducer/activator of molecular oxygen. In aqueous systems, it is known [24] that flavins could bring about a reduction of molecular oxygen via two routes, depending upon the spin state (an adapted version is shown in Figure 5a)-

- a slow reaction with triplet oxygen (E°'∼−160 mV), giving superoxide &
- a fast reaction with singlet oxygen (first electron transfer E°'∼+640 mV), giving peroxide.

The auto-dismutation of superoxide, originally produced by CPR, could also account for a significant part of peroxide formed in CYP+CPR reaction milieu. Sometimes, a higher peroxide concentration in the presence of CYPs could be due to- (1) the ability of the heme metal atom to facilitate the peroxide generation by the reducible metal center serving as a superoxide dismutase & (2) a lesser probable route could be that the metal center could catalyze the endothermic process of singlet oxygen generation, which could react faster with CPR giving peroxide. The presence of singlet oxygen in such reaction mixtures have been documented earlier [20], [25] and stressed on recently by Hayashi et al [26]. Both these processes of peroxide generation could perhaps be more efficient if the iron is in high spin state, like many native CYP2E1.

In 1994, Vincent Massey had said [22]- “Because of the higher redox potential of the couple O_2_/H_2_O_2_ *(E_m7_* =  +270 mV) versus that of oxidized/reduced flavin *(E_m_* = −209 mV), the overall 2-electron oxidation reaction, Fl_red_ H_2_+O_2_ → Fl_ox_+H_2_O_2_, is essentially irreversible.” However, we have seen here that the flavoenzyme CPR could also indulge in the very same peroxide depleting activity as its more illustrious heme-counterparts. In the heme-based classical peroxidase/catalase enzyme systems, the activation with peroxide leads to a two electron jump into an oxidized enzyme complex, which could undergo either a ‘two-steps single-electron’ or a ‘single-step two-electrons’ pathway, for the enzyme to recycle. In CPR, the activation by peroxide is highly unlikely to yield a two-electron reduced CPR, owing to the reason quoted by Massey. But it could lead to a one-electron reduced semiquinone enzyme and a diffusible radical by-product. This reaction (H_2_O_2_ → O_2_^−^+2H^+^+e^−^) has a redox potential of −890 mV. This low value makes the reaction quite favorable with the redox ranges of the two flavins of CPR, which has standard potentials ranging from −110 mV to −365 mV for its various one electron reactions [27]. The first one electron transfer phase of the proposed mechanism (which is shown in Figure 5b) in this work is in accordance with the route already postulated for other flavoenzymes, like nitropropane dioxygenase [23], [28]. Reaction of the semiquinone with a second molecule of peroxide would generate yet another diffusible radical and water, giving back the native enzyme. Both the liberated diffusible free radicals could further react with peroxide, leading to the depletion of the same. In our preliminary studies, the spectral signature of the reaction mixture of micromolar CPR with millimolar peroxide gave implications of a semiquinone species (absorbance at 455 nm was relatively unchanging but that at 366 nm increased). Stopped-flow spectroscopic characterization of the dynamically produced reaction intermediates would afford meaningful insights to greater details of the mechanism.

**Figure 6:**
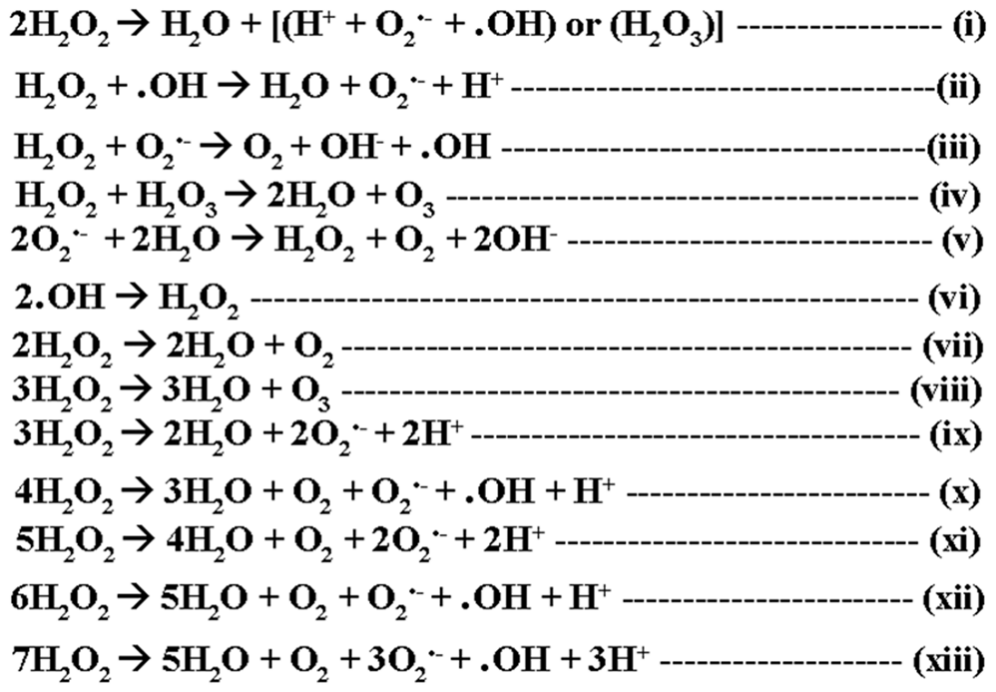
Probable DROS reactions that could be envisaged to occur in milieu.

The probable reactions that could occur in the milieu are shown in Figure 6. The equation (i) would be the basic CPR catalyzed cycle. Equations (ii) through (iv) could be the original enzymatic catalysis products' reactions with the excess peroxide. Equations (v) and (vi) would be anticipated to occur in water spontaneously. Therefore, net stoichiometric equation for peroxide depletion could be any one of the equations (vii) through (x) and further combinations thereof, exemplified by (xi) through (xiii). It can now be understood that labeled molecular oxygen could also lead to the production of labeled water (a phenomena which was erstwhile considered to be a proof for the hypothesis that Compound I formed water, in the presence of poor substrates!) from CPR's oxygen activation and peroxide depleting activities, without the need for heme-center involvement. In this investigation, we observed that-

- Oxygen evolution was much lower for a corresponding amount of peroxide depleted; which underplays the catalase type equation (vii).
- Incorporation of indigo sulfonate (a reactant probe for ozone) gave only a marginal activity in the initial time frame; which underplays equation (viii).
- CPR did not show a Michaelis-Menten hyperbolic relation with peroxide as substrate.
- Radical intermediates are involved in the reaction.
- Exact stoichiometry could not be established even when reaction compositions/environments were varied only on a very low note.

Therefore, we could infer that the outcome of the overall CPR catalyzed reaction can be seen as a combination of the basic enzymatic and several non-enzymatic reactions within the milieu (as shown in Figure 6). The equations (ix) through (xiii) and the likes of higher order equations could explain lower oxygen output. The outcome would also be expected to vary significantly with slight variations in conditions, due to the number of competing reactions involved. Thus, the time-based variations of peroxide in milieu and loss of redox equivalents in the overall stoichiometry of CYP+CPR reactions could be better explained by the DROS regulating role of CPR.

To conclude, this communication offers an evidence-based alternate viewpoint to the established paradigm in CYP+CPR mechanism and reports for the first time, a peroxide depleting function for a flavoenzyme. The presence of CPR would lead to small amounts of superoxide in the milieu and when this keeps accruing and turning to peroxide, CPR also sees to it that the peroxide formed is recycled to water. This would, however, be a radical mediated and potentially chaotic process. But the presence of peroxide itself acts as a ‘temporary cushion’ against the radicals. This is quite analogous to the reaction microenvironment of chloroperoxidase, in which the reaction system consumes the chlorinating intermediate generated in the absence of a suitable final acceptor [29]. We have also recently shown that diffusible radical mediated reactions play key roles in determining the overall kinetic outcomes in CYP mediated reactions [30]. The newly established reactivity of CPR would also drain the cell of redox reserves. Though these are negative aspects for the order cum economy seeking cellular machinery, their presence or roles may be unavoidable. From the information gained in this work, we can now understand the low distribution density of CPR and the growth-inhibitory effects observed during over-expression in several strains of CPR transformed cells. Also, the loss of redox equivalents in CYP+CPR reactions can now be accounted for by the DROS regulating roles of CPR. Currently, the uncoupling (loss of redox equivalents and generation of DROS) is considered to be an attribute of CYPs and drugs are modeled for lowering the ‘uncoupling’ on the basis of the erstwhile hypothesis [14]. Our work points out that such consideration and approach could lead to erroneous outcomes.

## Materials and Methods

Purified human CYP2C9 and CPR were lab preparations [31] and pure lyophilized HRP, met-myoglobin and catalase were procured from Sigma. Concentration of CYP was determined by dithionite reduction followed by CO binding [32]. Other hemoproteins' concentrations were determined by calculation using a molar extinction coefficient of ∼10^5^ for the Soret band. Concentration of CPR was determined under oxidizing conditions with excess potassium ferricyanide, using a molar extinction coefficient of 21,200 for CPR at 455 nm. All reactions were carried out in aerated open vials at 37°C (unless otherwise stated). A commercial hydrogen peroxide stock solution (∼8.8 M) was diluted and calibrated around several millimolar ranges to determine the absolute concentration by titanium oxalate complexation method [33]. A small aliquot of a known peroxide stock was dispensed into the reactions. Concentration of peroxide remaining in the reaction mixture was estimated by the Peroxoquant method of Pierce Chemicals [34]. The linear standard plot gave an R^2^ value >0.99 for OD at 560 nm for the colored product. NADPH was determined by spectrophotometry at 340 nm using an extinction coefficient of 6220 M^−1^cm^−1^. Peroxide is taken as the index of DROS in milieu because the thermodynamic and equilibrium considerations favor peroxide over superoxide by many folds [35], [36]. Evolution of oxygen in small volumes of enzyme reactions was monitored by a suitable microelectrode from Lazar Research Labs, USA. Other pertinent details of methods are given below-

### Tracing peroxide production in pure reconstituted systems of CYP and CPR

A control of 100 nM CYP with 200 µM NADPH gave no observable peroxide at 15 min and depleted NADPH at the rate of ∼0.2 µM/min, which is only slightly higher than the auto-degradation rate of NADPH under these conditions. Depletion rate of NADPH (practically zeroth order within the conversions reported herein) was determined from the slope of linear fit of 340 nm OD at 16, 26 and 36 minutes. The linear fit had R^2^ values greater than 0.997. Rates for NADPH consumption had less than 5% SEM. For the NADH reaction, the initial concentrations of components, when present, were- (NADH)  = 150 µM. (CPR)  = 25 or 100 or 500 nM, (CYP2C9)  = 100 nM, (diclofenac)  = 100 µM.

### Effect of a radical scavenger in CPR mediated peroxide depletion

The reaction mixture had an initial concentration of (CPR)  = 250 nM and (H_2_O_2_)  = 500 µM taken in 100 mM phosphate buffer (pH 7.4, 28°C), which also contained DLPC/lecithin vesicles ∼500 µg/ml. L-ascorbic acid palmitate (AAP), when present, was at a concentration of 1 mM. 50 µl of the reaction sample was added to 600 µl of peroxoquant reagent and incubated for 30 minutes and the samples were read at 560 nm.

### Tracing oxygen evolution in peroxide depletion reactions

The overall spread of electrode potential readings varied from a low reading of ∼190 mV for initial values for CPR containing mixture to a high value of ∼350 mV for the later time values (∼1 minute) of catalase reaction. These spread in readings were extrapolated to dissolved oxygen concentrations, using standard values accounting for the temperature and altitude compensation. Temperature and pH were 29°C and 7.1 respectively. A relatively high initial concentration of 10 mM peroxide was necessary because the oxygen evolved with micromolar concentrations were too low to be measured by the electrode. Concentration of enzymes was ∼50 nM. The X-axis is broken to show initial and later time oxygen evolution profile.
